# The effects of the attributable fraction and the duration of symptoms on burden estimates of influenza‐associated respiratory illnesses in a high HIV prevalence setting, South Africa, 2013‐2015

**DOI:** 10.1111/irv.12529

**Published:** 2018-02-01

**Authors:** Stefano Tempia, Sibongile Walaza, Jocelyn Moyes, Adam L. Cohen, Claire von Mollendorf, Meredith L. McMorrow, Sarona Mhlanga, Florette K. Treurnicht, Marietjie Venter, Marthi Pretorius, Orienka Hellferscee, Nicole Wolter, Anne von Gottberg, Arthemon Nguweneza, Johanna M. McAnerney, Halima Dawood, Ebrahim Variava, Shabir A. Madhi, Cheryl Cohen

**Affiliations:** ^1^ Influenza Division Centers for Disease Control and Prevention Atlanta GA USA; ^2^ Influenza Program Centers for Disease Control and Prevention Pretoria South Africa; ^3^ Centre for Respiratory Diseases and Meningitis National Institute for Communicable Diseases of the National Health Laboratory Service Johannesburg South Africa; ^4^ School of Public Health Faculty of Health Sciences University of the Witwatersrand Johannesburg South Africa; ^5^ Global Immunization Monitoring and Surveillance Team, Expanded Programme on Immunization Department of Immunization, Vaccines and Biological World Health Organization Geneva Switzerland; ^6^ Centre for Viral Zoonoses Department of Medical Virology University of Pretoria Pretoria South Africa; ^7^ Tshwane Academic Division National Health Laboratory Service Pretoria South Africa; ^8^ School of Pathology Faculty of Health Sciences University of the Witwatersrand Johannesburg South Africa; ^9^ Department of Medicine Pietermaritzburg Metropolitan Hospital Pietermaritzburg South Africa; ^10^ Department of Medicine University of KwaZulu‐Natal Pietermaritzburg South Africa; ^11^ Department of Medicine Klerksdorp‐Tshepong Hospital Complex Klerksdorp South Africa; ^12^ Department of Medicine Faculty of Health Sciences University of the Witwatersrand Johannesburg South Africa; ^13^ Perinatal HIV Research Unit University of the Witwatersrand Johannesburg South Africa; ^14^ Medical Research Council Respiratory and Meningeal Pathogens Research Unit University of the Witwatersrand Johannesburg South Africa; ^15^ Department of Science and Technology/National Research Foundation: Vaccine Preventable Diseases University of the Witwatersrand Johannesburg South Africa

**Keywords:** attributable fraction, HIV, influenza, influenza‐like illness, rates, severe respiratory illness, South Africa, symptom duration

## Abstract

**Background:**

The attributable fraction of influenza virus detection to illness (INF‐AF) and the duration of symptoms as a surveillance inclusion criterion could potentially have substantial effects on influenza disease burden estimates.

**Methods:**

We estimated rates of influenza‐associated influenza‐like illness (ILI) and severe acute (SARI‐10) or chronic (SCRI‐10) respiratory illness (using a symptom duration cutoff of ≤10 days) among HIV‐infected and HIV‐uninfected patients attending 3 hospitals and 2 affiliated clinics in South Africa during 2013‐2015. We calculated the unadjusted and INF‐AF‐adjusted rates and relative risk (RR) due to HIV infection. Rates were expressed per 100 000 population.

**Results:**

The estimated mean annual unadjusted rates of influenza‐associated illness were 1467.7, 50.3, and 27.4 among patients with ILI, SARI‐10, and SCRI‐10, respectively. After adjusting for the INF‐AF, the percent reduction in the estimated rates was 8.9% (rate: 1336.9), 11.0% (rate: 44.8), and 16.3% (rate: 22.9) among patients with ILI, SARI‐10, and SCRI‐10, respectively. HIV‐infected compared to HIV‐uninfected individuals experienced a 2.3 (95% CI: 2.2‐2.4)‐, 9.7 (95% CI: 8.0‐11.8)‐, and 10.0 (95% CI: 7.9‐12.7)‐fold increased risk of influenza‐associated illness among patients with ILI, SARI‐10, and SCRI‐10, respectively. Overall 34% of the estimated influenza‐associated hospitalizations had symptom duration of >10 days; 8% and 44% among individuals aged <5 and ≥5 years, respectively.

**Conclusion:**

The marginal differences between unadjusted and INF‐AF‐adjusted rates are unlikely to affect policies on prioritization of interventions. HIV‐infected individuals experienced an increased risk of influenza‐associated illness and may benefit more from annual influenza immunization. The use of a symptom duration cutoff of ≤10 days may underestimate influenza‐associated disease burden, especially in older individuals.

## 1 INTRODUCTION

Influenza virus infections cause substantial morbidity and mortality globally, in particular among individuals aged <5 and ≥65 years and persons with underlying medical conditions, including HIV infection.^1^, ^2^, ^3^, ^4^, ^5^, ^6^

The World Health Organization (WHO) highlighted that there is a need for influenza disease burden estimates especially from low‐ and middle‐income countries.^7^ Such estimates would enable governments to make informed evidence‐based decisions when allocating scarce resources and planning intervention strategies to limit the impact and spread of the disease. In addition, national estimates would contribute to the global understanding of the burden of influenza‐associated severe illness and inform global public health priorities.

Whereas estimates of influenza‐associated mortality have been generated mainly from ecological studies,^1^, ^5^, ^6^, ^8^, ^9^, ^10^ the majority of estimates of influenza‐associated hospitalization and outpatient consultation have been obtained from laboratory‐confirmed influenza surveillance conducted at selected sentinel sites.^2^, ^3^, ^4^, ^11^ In recent years, such surveillance systems have relied on the use of polymerase chain reaction (PCR) assays for the detection of influenza viruses and the use of standard case definitions for the identification and inclusion of patients in the surveillance programs.

Although the wide use of PCR assays has increased diagnostic and surveillance capacity, establishing a clinical association between pathogen detection and illness remains challenging. A study conducted in South Africa suggested that the attributable fraction (AF) of influenza virus detection to mild and severe respiratory illness (referred to as INF‐AF hereafter) varies by age group and HIV serostatus.^12^ However, the effect of the INF‐AF on influenza disease burden estimates has not been fully investigated.

In addition, the WHO recommends the implementation of influenza sentinel surveillance using standard case definitions among outpatients with influenza‐like illness (ILI) and inpatients with severe acute respiratory illness (SARI).^13^ The WHO recommended case definitions were updated in 2012 to include, among other changes, patients with symptoms duration of ≤10 days (compared to ≤7 days as previously recommended) with the aim to increase sensitivity of influenza virus detection.^13^ Nonetheless, the effect of symptom duration on influenza disease burden estimates among individuals of different age groups and HIV serostatus is not fully understood.

A better understanding of the effects of the INF‐AF and the duration of symptoms as a surveillance inclusion criterion on influenza disease burden estimates could provide insights on the vaccine‐preventable fraction of illness and contextualize the use of recommended surveillance case definitions for the estimation of disease burden.

We aimed to assess the effects of the INF‐AF and the duration of symptoms on influenza disease burden estimates among HIV‐infected and HIV‐uninfected patients of different age groups presenting with ILI, SARI, and severe chronic respiratory illness (SCRI) in South Africa from January 2013 through December 2015.

## 2 METHODS

### 2.1 Description of the surveillance programs

#### 2.1.1 Severe respiratory illness surveillance

We conducted active prospective hospital‐based surveillance for SARI and SCRI at 3 public hospitals in 2 provinces serving a population of 456 975 people in 2015 [Edendale Hospital in a peri‐urban area of KwaZulu‐Natal Province, and Klerksdorp and Tshepong Hospitals (the Klerksdorp‐Tshepong Hospital Complex, KTHC) in a peri‐urban area of North West Province] from January 2013 through December 2015.

A case of SARI was defined as a hospitalized person who had illness onset within 10 days of admission (referred to as SARI‐10 hereafter) and who met age‐specific clinical inclusion criteria. A case in children aged 2 days to <3 months included any hospitalized patient with diagnosis of suspected sepsis or physician‐diagnosed acute lower respiratory tract infection irrespective of signs and symptoms. A case in children aged 3 months to <5 years included any hospitalized patient with physician‐diagnosed acute lower respiratory tract infection, including bronchitis, bronchiolitis, pneumonia, and pleural effusion. A case in individuals aged ≥5 years included any hospitalized patient presenting with manifestation of acute lower respiratory tract infection with temperature ≥38°C or history of fever and cough.^13^

A case of SCRI was defined as a hospitalized person who had illness onset >10 days (referred to as SCRI‐10 hereafter) and who met the above age‐specific clinical inclusion criteria.

#### 2.1.2 Influenza‐like illness surveillance

We conducted prospective surveillance for cases presenting with ILI at two outpatient clinics (Edendale Gateway Clinic, KwaZulu‐Natal Province, and Jouberton Clinic, North West Province) located in the same catchment area to the above‐mentioned hospitals over the same study period.

An ILI case was defined as an outpatient of any age presenting with either temperature ≥38°C or history of fever and cough of duration of ≤10 days.

#### 2.1.3 Study procedures

The procedures of these surveillance programs have been previously described.^4^, ^12^, ^14^, ^15^, ^16^ In brief, study staff completed case report forms for all enrolled ILI, SARI‐10, and SCRI‐10 cases. Referral to hospital was recorded for all enrolled ILI cases. ILI cases that were referred to hospital were excluded from the analysis. Numbers of patients meeting the ILI, SARI‐10, and SCRI‐10 case definitions and numbers enrolled were collected throughout the study period. Outpatient care prior to hospitalization was also recorded for enrolled SARI‐10 and SCRI‐10 cases.

Age‐ and year‐specific population denominators were obtained from projections of 2011 census data,^17^ while age‐ and year‐specific HIV prevalence in the study population was obtained from the projections of the THEMBISA Model.^18^

### 2.2 Laboratory procedures

Respiratory specimens (ie, nasopharyngeal aspirates for children aged <5 years and nasopharyngeal and oropharyngeal swabs from persons aged ≥5 years) were collected from all enrolled patients (ILI, SARI‐10, and SCRI‐10 cases), placed in universal transport medium, stored at 4‐8°C, and transported to the National Institute for Communicable Diseases (NICD) within 72 hours of collection for testing. Specimens were tested for the presence of 10 respiratory viruses (influenza A and B viruses; parainfluenza virus types 1, 2, and 3; respiratory syncytial virus; adenovirus; rhinovirus; human metapneumovirus; and enterovirus) using a multiplex real‐time reverse transcription PCR assay.^19^

### 2.3 Determination of HIV status

HIV results were obtained from a combination of two sources: (i) patient clinical records when available and (ii) for consenting patients, a dried blood spot was tested at NICD. Testing included HIV enzyme‐linked immunosorbent assay (ELISA) for patients aged ≥18 months and PCR for children aged <18 months if the ELISA was reactive.

### 2.4 Statistical analysis

#### 2.4.1 Unadjusted and attributable fraction‐adjusted rates of influenza‐associated respiratory hospitalizations and outpatient consultations

The details of the calculations of rates are provided in Supplementary Material. In brief, we estimated the overall and age‐specific rates of influenza‐associated SARI‐10 hospitalizations per 100 000 population using the number of SARI‐10 hospitalizations, adjusting for non‐enrollment (refusal to participate and non‐enrollment during weekends), healthcare‐seeking behavior in the surveyed community,^20^, ^21^ and the INF‐AF during 2013‐2015. The INF‐AF was obtained from published estimates derived from the same sentinel sites and study period.^12^

The same approach was used to estimate the rates of influenza‐associated SCRI‐10 hospitalizations.

We estimated the rates of influenza‐associated ILI consultations per 100 000 population from the estimated SARI‐10 rates using the proportion of SARI‐10 cases that sought care at primary healthcare facilities prior to hospitalization and the proportion of ILI cases referred to hospital from ILI surveillance data.

For all calculations, we assumed that the influenza detection rate among individuals tested and not tested was the same within syndromes and age groups.

For all estimates the unadjusted and AF‐adjusted rates, rate difference and percent reduction (PR) between unadjusted and AF‐adjusted rates were reported.

Because our SARI‐10 case definition among children aged <5 years differed from those recommended by WHO (ie, any hospitalized patient presenting with manifestation of acute lower respiratory tract infection with temperature ≥38°C or history of fever and cough), we also calculated the proportion of any influenza‐positive respiratory hospitalizations that met the WHO case definition in this age group.

#### 2.4.2 HIV‐stratified rates and risk of HIV infection on influenza‐associated respiratory illness

We obtained the HIV‐stratified rates of influenza‐associated ILI, SARI‐10, and SCRI‐10 by multiplying the total number of influenza cases by the age‐specific HIV prevalence among influenza‐positive cases divided by the HIV serostatus‐specific population at risk.

Subsequently, we estimated the HIV‐stratified AF‐adjusted rates of influenza‐associated ILI, SARI‐10, and SCRI‐10 by multiplying the observed rates by the estimated age‐ and HIV serostatus‐specific INF‐AF.^12^

Age‐specific and overall age‐adjusted relative risk (RR) for influenza‐associated ILI, SARI‐10, and SCRI‐10 among HIV‐infected and HIV‐uninfected persons was estimated using log‐binomial regression. This was done for both unadjusted and AF‐adjusted rates.

#### 2.4.3 Sensitivity analysis of the impact of duration of symptoms on rates of influenza‐associated hospitalizations

We implemented the above analysis after reclassifying the SARI and SCRI cases using a duration of symptoms cutoff of 7 days (referred to as SARI‐7 and SCRI‐7 hereafter). This classification was in accordance with practices previously recommended by WHO for global influenza surveillance. This analysis was implemented to assess the changes in rates of influenza‐associated hospitalization among SARI cases as defined in previously published literature in Africa,^22^ including South Africa^4^ in comparison with the current case definition that uses a symptom duration cutoff of 10 days.

We obtained the 95% confidence intervals (CI) for all estimated rates using bootstrap resampling of all datasets used in the estimation over 1000 replications. This included the proportion of SARI and SCRI cases enrolled, the influenza virus percentage positive within each syndrome, the INF‐AF, and the healthcare utilization behavior obtained from the HUS. The lower and upper limits of the 95% CI were the 2.5th and 97.5th percentile of the estimated values from the bootstrapped datasets, respectively.

### Ethical approval

The SARI and SCRI protocols were approved by the University of the Witwatersrand Human Research Ethics Committee (HREC) and the University of KwaZulu‐Natal Human Biomedical Research Ethics Committee (BREC) protocol numbers M081042 and BF157/08, respectively. The ILI protocol was approved by BREC protocol number (BREC BF 080/12). This surveillance was deemed non‐research by the US Centers for Disease Control and Prevention (non‐research determination number: 2012‐6197).

## 3 RESULTS

### 3.1 Study population

During 2013‐2015, we enrolled 8422 individuals of which 8030 (95.3%) had known age and available influenza and HIV results and were included for further analysis. Of these 3662 (45.6%) were ILI cases, 2739 (34.1%) were SARI‐10 cases (SARI‐7: 1736; 21.6%) and 1629 (20.3%) were SCRI‐10 cases (SCRI‐7: 2632; 32.8%). The median duration of illness was 2 days (range 0‐10 days) among patients with ILI, 3 days (range 0‐10 days) among patients with SARI‐10, and 16 days (range 11‐37 days) among patients with SCRI‐10.

Children aged <5 years accounted for 35.6% (1302/3662) of ILI cases, 55.5% (1520/2739) of SARI‐10 cases (SARI‐7: 79.0%; 1371/1736), and 6.7% (109/1629) of SCRI‐10 cases (SCRI‐7: 9.8%; 258/2374). The HIV prevalence was 25.2% (923/3662) among ILI cases, 37.3% (1022/2739) among SARI‐10 cases (SARI‐7: 21.8%; 378/1736), and 72.1% (1175/1629) among SCRI‐10 cases (SRCI‐7: 69.1%; 1819/2632) (*P *< .001). Across syndromes, the HIV prevalence was lowest among infants aged <1 year [ILI: 1.7% (8/465); SARI‐10: 8.9% (86/963); SARI‐7: 8.4% (75/894); SCRI‐10: 17.1% (12/70); SCRI‐7: 16.5% (23/139)] and highest among persons aged 25‐44 years [ILI: 59.4% (584/983); SARI‐10: 89.9% (514/572); SARI‐7: 89.1% (147/165); SCRI‐10: 91.6% (716/782); SCRI‐7: 91.1% (1083/1189)]. The HIV prevalence by age group and syndrome is provided in Figure S1.

**Figure 1:**
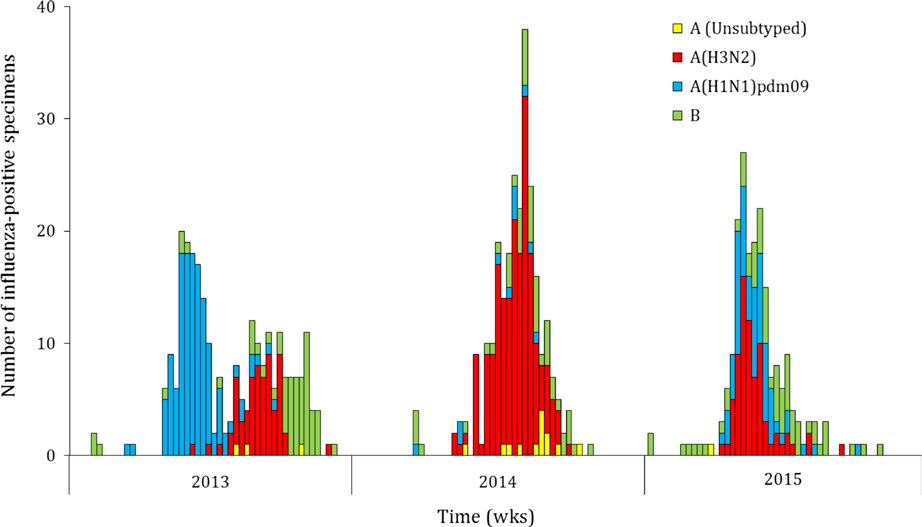
Weekly number of influenza‐positive specimens by type and subtype among outpatients with influenza‐like illness and inpatients with severe acute or chronic respiratory illness, Klerksdorp and Pietermaritzburg, South Africa, 2013‐2015

Influenza viruses were detected in 8.6% (689/8030) of specimens. Of these, 45.7% (315/689) were influenza A(H3N2), 28.6% (197/689) were influenza A(H1N1)pdm09, 2.0% (14/689) were influenza A not subtyped, and 23.7% (163/689) were influenza B (Figure 1). Influenza viruses were detected in 13.4% (489/3662) of ILI cases, 5.1% (139/2739) of SARI‐10 cases (SARI‐7: 4.8%; 83/1736), and 3.7% (61/1629) of SCRI‐10 cases (SCRI‐7: 4.4%; 117/2632) (*P* < .001). Among influenza‐positive children aged <5 years hospitalized with any severe respiratory illness 68.1% (62/91) met the WHO SARI case definition; 57.5% (27/47) among infants aged <1 year and 79.6% (35/44) among children aged 1‐4 years, respectively.

### 3.2 Effects of the INF‐AF on burden estimates of influenza‐associated respiratory illnesses

#### 3.2.1 Effects of the INF‐AF on estimated rates

**Figure 2:**
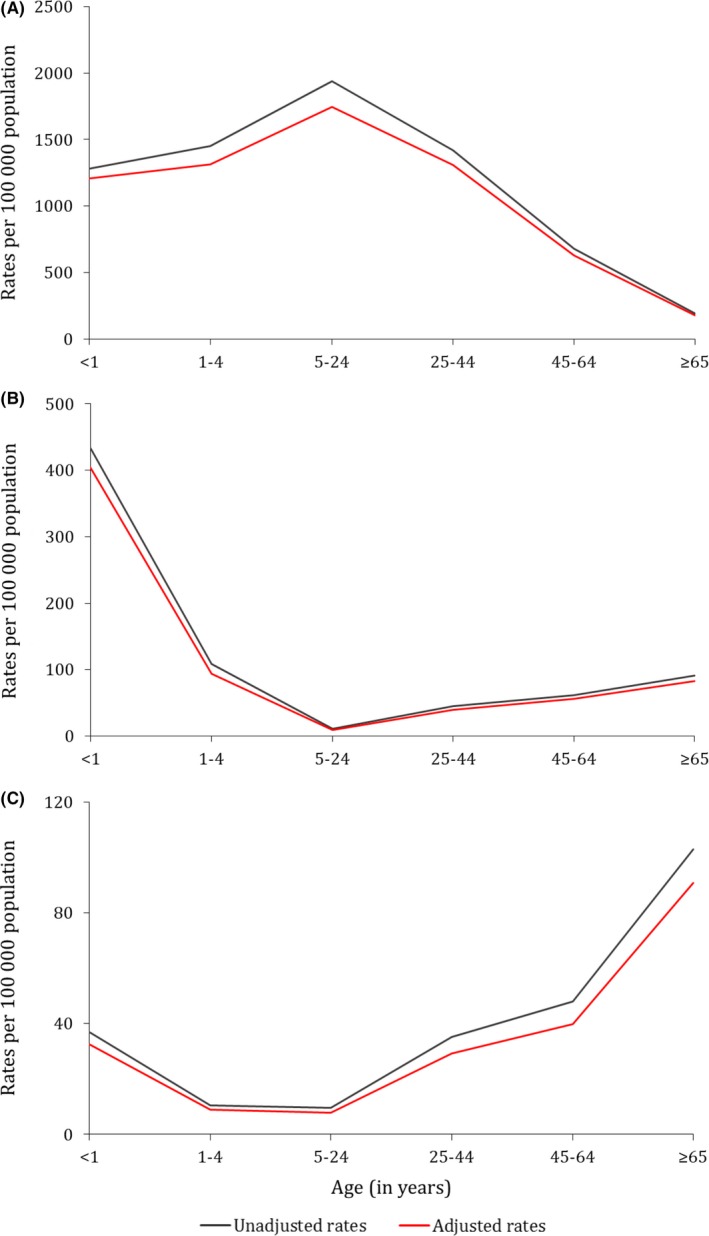
Overall unadjusted and attributable fraction‐adjusted mean annual influenza‐associated respiratory illness rates by age group, Klerksdorp and Pietermaritzburg, South Africa, 2013‐2015. (A) Influenza‐like illness; (B) severe acute respiratory illness (symptom duration ≤10 days); (C) severe chronic respiratory illness (symptom duration >10 days)

During the study period, the overall unadjusted mean annual influenza‐associated illness rates per 100 000 population were 1467.7 among ILI cases, 50.3 among SARI‐10 cases, and 27.4 among SCRI‐10 cases (Table 1). The mean annual unadjusted influenza‐associated illness rates per 100 000 population were highest in the 5‐24 year age group (1938.8) among ILI cases, in the <1 year age group (404.2) among SARI‐10 cases, and in the ≥65 year age group (91.0) among SCRI‐10 cases (Table 1 and Figure 2). After adjusting for the INF‐AF, the percent reduction (PR) from the overall unadjusted rates was 8.9% (AF‐adjusted rate: 1336.9), 11.0% (AF‐adjusted rate: 44.8) and 16.3% (AF‐adjusted rate: 22.9) among ILI, SARI‐10, and SCRI‐10 cases, respectively. The 95% CIs overlapped between the unadjusted and AF‐adjusted rates across syndromes, age groups, and HIV serostatus. Overall, the effects of the INF‐AF on the estimated rates were higher among HIV‐uninfected (Table 2) than HIV‐infected (Table 3) individuals across syndromes (PR among ILI cases: HIV‐infected 4.9% vs HIV‐uninfected 10.3%; PR among SARI‐10 cases: HIV‐infected 9.4% vs HIV‐uninfected 12.3%; PR among SCRI‐10 cases: HIV‐infected 15.5% vs HIV‐uninfected 17.5%).

**Table 1:** Estimated mean annual rates of influenza‐associated influenza‐like‐illness, severe acute respiratory illness (symptom duration ≤10 days) and severe chronic respiratory illness (symptom duration >10 days) among any patient (irrespective of HIV serostatus), Klerksdorp and Pietermaritzburg, South Africa, 2013‐2015

Age group (in years)	Influenza‐associated respiratory illness rates^a^ (95% CI)		
	Unadjusted^b^	AF‐adjusted^c^	Rate difference^d^ (% reduction)
Influenza‐like illness			
<1	1282.1 (1158.7‐1416.5)	1207.0 (1087.1‐1337.2)	‐75.1 (5.9)
1‐4	1452.8 (1386.3‐1522.4)	1313.2(1249.6‐1389.0)	‐139.6 (9.6)
5‐24	1938.8 (1823.6‐2024.6)	1747.9 (1712.5‐1883.6)	‐190.9 (9.9)
25‐44	1471.1 (1381.2‐1453.6)	1308.7 (1274.4‐1443.9)	‐108.4 (7.6)
45‐64	678.8 (643.2‐715.9)	629.5 (595.0‐665.1)	‐49.3 (7.3)
≥65	193.3 (157.9‐236.0)	178.0 (143.9‐218.9)	‐15.3 (7.9)
<5	1418.7 (1359.3‐1479.5)	1292.0 (1235.6‐1360.3)	‐126.7 (8.9)
≥5	1473.9 (1452.2‐1495.7)	1342.6 (1321.9‐1463.4)	‐131.3 (8.9)
All	1467.7 (1447.3‐1488.2)	1336.9 (1317.5‐1456.5)	‐130.8 (8.9)
Severe acute respiratory illness (SARI‐10)			
<1	433.6 (362.3‐513.6)	404.2 (335.4‐481.5)	‐29.4 (6.8)
1‐4	108.5 (91.2‐129.1)	94.2 (77.8‐113.1)	‐14.3(13.2)
5‐24	10.8 (8.2‐14.0)	9.0 (6.6‐11.9)	‐1.8(16.2)
25‐44	45.3 (39.2‐53.3)	40.0 (34.1‐46.4)	‐5.3(11.6)
45‐64	62.1 (51.6‐74.0)	55.7 (45.7‐66.9)	‐6.4 (10.4)
≥65	91.0 (67.0‐121.3)	82.9 (60.4‐112.4)	‐8.1(8.9)
<5	173.5 (153.4‐195.9)	156.1 (136.8‐177.1)	‐17.4 (10.0)
≥5	34.7 (31.4‐38.2)	30.7 (27.7‐34.0)	‐4.0 (11.6)
All	50.3 (46.6‐54.2)	44.8 (41.3‐48.5)	‐5.5 (11.0)
Severe Chronic respiratory illness (SCRI‐10)			
<1	37.0 (18.0‐64.6)	32.6 (15.7‐60.3)	‐4.4 (11.9)
1‐4	10.5 (5.7‐18.2)	8.9 (4.5‐16.1)	‐1.6 (15.5)
5‐24	9.6 (7.1‐12.5)	7.8 (5.7‐10.6)	‐1.8 (19.4)
25‐44	35.2 (29.7‐41.3)	29.3 (24.4‐35.1)	‐5.9 (16.6)
45‐64	48.1 (38.9‐58.7)	39.8 (31.3‐49.3)	‐8.3 (17.4)
≥65	102.9 (77.0‐134.5)	90.8 (67.0‐49.3)	‐12.1 (11.7)
<5	15.8 (10.1‐23.4)	13.6 (8.5‐21.0)	‐2.2 (13.8)
≥5	28.9 (25.9‐32.1)	24.1 (21.4‐27.0)	‐4.8 (16.5)
All	27.4 (24.7‐30.3)	22.9 (20.4‐25.6)	‐4.5 (16.3)

CI, confidence intervals; AF, attributable fraction.

a Rates expressed per 100,000 population.

b Estimated rates without adjustment for the attributable fraction.

c Estimated rates adjusted by the attributable fraction.

d Unadjusted minus attributable fraction‐adjusted rates.

**Table 2:** Estimated mean annual rates of influenza‐associated influenza‐like‐illness, severe acute respiratory illness (symptom duration ≤10 days) and severe chronic respiratory illness (symptom duration >10 days) among HIV‐uninfected patients, Klerksdorp and Pietermaritzburg, South Africa, 2013‐2015

Age group (in years)	Influenza‐associated respiratory illness rates^a^ (95% CI)		
	Unadjusted^b^	AF‐adjusted^c^	Rate difference^d^ (% reduction)
Influenza‐like illness			
<1	1280.5 (1156.4‐1414.9)	1204.9 (1084.1‐1335.0)	‐75.6 (5.9)
1‐4	1450.4 (1383.2‐1520.5)	1309.7 (1245.6‐1396.0)	‐140.7 (9.7)
5‐24	1883.4 9 (1845.4‐1921.8)	1691.3 (1655.4‐1867.8)	‐191.1 (10.2)
25‐44	821.4 (788.8‐854.9)	720.4 (690.0‐791.8)	‐101.0 (12.3)
45‐64	521.9 (487.5‐558.6)	476.5 (443.2‐511.2)	‐45.4 (8.7)
≥65	186.4 (150.3‐228.2)	171.1 (136.0‐210.5)	‐15.3 (8.2)
<5	1416.1 (1356.8‐1477.9)	1288.5 (1231.6‐1387.1)	‐127.6 (9.0)
≥5	1265.6 (1243.7‐1287.8)	1132.9 (1112.2‐1253.9)	‐132.7 (10.5)
All	1289.2 (1268.6‐1310.2)	1156.8 (1137.3‐1276.6)	‐132.4 (10.3)
**Severe acute respiratory illness (SARI‐10)**			
<1	431.9 (362.5‐514.5)	402.5 (335.3‐482.1)	‐29.4 (6.8)
1‐4	101.6 (84.6‐121.6)	87.9 (71.7‐106.1)	‐13.7 (13.5)
5‐24	6.6 (5.6‐9.3)	5.2 (3.4‐7.6)	‐1.4 (21.4)
25‐44	8.9 (5.8‐13.1)	6.8 (4.2‐10.6)	‐2.1 (24.4)
45‐64	17.0 (11.0‐24.4)	14.5 (9.0‐21.4)	‐2.5 (14.8)
≥65	75.2 (53.8‐104.5)	68.4 (47.2‐95.2)	‐6.8 (9.0)
<5	168.1 (147.9‐190.0)	151.3 (132.3‐172.3)	‐16.8 (10.0)
≥5	12.3 (10.3‐14.7)	10.3 (8.3‐12.4)	‐2.0 (16.8)
All	32.7 (29.5‐36.1)	28.7 (25.7‐31.9)	‐4.0 (12.3)
**Severe Chronic respiratory illness (SCRI‐10)**			
<1	36.5 (18.2‐65.1)	32.2 (15.9‐60.8)	‐4.3 (11.9)
1‐4	9.3 (4.5‐16.4)	7.8 (3.4‐14.3)	‐1.5 (16.1)
5‐24	7.4 (5.2‐10.2)	5.8 (3.9‐8.3)	‐1.6 (21.2)
25‐44	4.0 (2.1‐7.2)	2.8 (1.2‐5.4)	‐1.2 (30.5)
45‐64	19.8 (13.6‐28.0)	16.2 (10.5‐23.6)	‐3.6 (18.4)
≥65	88.5 (64.0‐118.3)	77.9 (55.5‐106.8)	‐10.6 (12.0)
<5	14.8 (9.2‐22.2)	12.7 (7.6‐19.8)	‐2.1 (14.0)
≥5	12.4 (10.3‐14.8)	10.2 (8.3‐12.3)	‐2.2 (18.1)
All	12.8 (10.8‐15.0)	10.5 (98.7‐12.6)	‐2.3 (17.5)

CI, confidence intervals; AF, attributable fraction.

a Rates expressed per 100,000 population.

b Estimated rates without adjustment for the attributable fraction.

c Estimated rates adjusted by the attributable fraction.

d Unadjusted minus attributable fraction‐adjusted rates.

**Table 3:** Estimated mean annual rates of influenza‐associated influenza‐like‐illness, severe acute respiratory illness (symptom duration ≤10 days) and severe chronic respiratory illness (symptom duration >10 days) among HIV‐infected patients, Klerksdorp and Pietermaritzburg, South Africa, 2013‐2015

Age group (in years)	Influenza‐associated respiratory illness rates^a^ (95% CI)		
	Unadjusted^b^	AF‐adjusted^c^	Rate difference^d^ (% reduction)
Influenza‐like illness			
<1	1469.5 (412.8‐3879.4)	1446.0 (401.3‐3792.5)	‐23.5 (1.6)
1‐4	1583.4 (1112.2‐2198.5)	1497.9 (1038.6‐2095.8)	‐85.5 (5.4)
5‐24	2700.8 (2534.2‐2875.4)	2525.2 (2363.3‐2693.2)	‐175.6 (6.5)
25‐44	2784.4 (2693.2‐2877.5)	2659.1 (2570.1‐2750.2)	‐125.3 (4.5)
45‐64	1336.1 (1222.3‐1455.9)	1270.6 (1160.2‐1388.1)	‐65.5 (4.9)
≥65	408.6 (175.3‐898.6)	393.5 (137.2‐813.7)	‐15.1 (93.7)
<5	1574.6 (1131.3‐2156.3)	1495.4 (1064.6‐2064.8)	‐79.2 (5.0)
≥5	2521.5 (2452.3‐2592.1)	2397.3 (2329.9‐2466.2)	‐124.2 (4.9)
All	2465.4 (2398.1‐2534.3)	2343.9 (2278.4‐2411.2)	‐121.5 (4.9)
Severe acute respiratory illness (SARI‐10)			
<1	629.8 (91.7‐1736.6)	593.9 (89.3‐1694.2)	‐35.9 (5.7)
1‐4	474.5 (242.2‐868.2)	428.0 (211.5‐811.2)	‐46.5 (9.8)
5‐24	68.0 (44.6‐101.8)	61.6 (38.0‐91.9)	‐6.4 (9.4)
25‐44	128.7 (109.4‐149.6)	116.4 (98.5‐136.9)	‐12.9 (9.6)
45‐64	250.9 (204.3‐307.4	228.0 (183.3‐281.6)	‐22.9 (9.1)
≥65	583.2 (256.4‐1064.5)	533.6 (234.3‐1023.2)	‐49.6 (8.5)
<5	491.7 (245.4‐829.8)	446.2 (217.4‐779.2)	‐45.5 (9.3)
≥5	147.2 (130.8‐165.0)	133.4 (117.6‐150.2)	‐13.8 (9.4)
All	148.9 (132.4‐166.2)	134.9 (119.6‐151.9)	‐14.0 (9.4)
Severe Chronic respiratory illness (SCRI‐10)			
<1	90.6 (8.4‐178.5)	80.7 (7.2‐156.3)	‐9.9 (11.0)
1‐4	75.7 (10.6‐318.7)	66.9 (8.9‐302.7)	‐8.8 (11.7)
5‐24	40.2 (23.2‐68.2)	34.3 (17.1‐57.8)	‐5.9 (14.7)
25‐44	106.6 (89.1‐125.8)	90.2 (74.7‐108.7)	‐16.4 (15.4)
45‐64	166.7 (128.0‐212.3)	138.5 (103.1‐180.1)	‐28.2 (16.9)
≥65	550.4 (256.4‐1064.5)	493.7 (215.2‐982.1)	‐56.7 (10.3)
<5	77.4 (9.6‐286.0)	68.5 (8.3‐254.6)	‐8.9 (11.6)
≥5	11.7 (97.5‐127.3)	94.3 (81.1‐108.6)	‐17.4 (15.6)
All	109.3 (95.5‐124.5)	92.3 (79.6‐106.4)	‐17.0 (15.5)

CI, confidence intervals; AF, attributable fraction.

a Rates expressed per 100,000 population.

b Estimated rates without adjustment for the attributable fraction.

c Estimated rates adjusted by the attributable fraction.

d Unadjusted minus attributable fraction‐adjusted rates.

The effects of the INF‐AF on the estimated rates among SARI‐7 and SCRI‐7 cases (Table 4) were similar to those observed among SARI‐10 and SCRI‐10 cases (Table 1, 2, 3). The unadjusted and AF‐adjusted influenza‐associated rates among any hospitalized patient are provided in Table S1 and Figure S2.

**Table 4:** Estimated mean annual rates of influenza‐associated severe acute respiratory illness (symptom duration ≤7 days) and severe chronic respiratory illness (symptom duration >7 days), Klerksdorp and Pietermaritzburg, South Africa, 2013‐2015

Age group (in years)	Influenza‐associated respiratory illness rates^a^ (95% CI)								
	All			HIV‐infected			HIV‐uninfected		
	Unadjusted^b^	AF‐adjusted^c^	Rate difference^d^ (% reduc.)	Unadjusted^b^	AF‐adjusted^c^	Rate difference^d^ (% reduc.)	Unadjusted^b^	AF‐adjusted^c^	Rate difference^d^ (% reduc.)
Severe acute respiratory illness (SARI‐7)									
<1	371.6 (305.5‐445.7)	343.4 (281.7‐417.0)	−28.2 (7.6)	595.6 (6.3‐1524.1)	555.7 (5.7‐1436.2)	−39.9 (6.7)	369.7 (305.2‐446.0)	341.6 (278.2‐413.4)	−28.1 (7.6)
1‐4	98.8 (82.2‐118.4)	85.3 (69.6‐103.2)	−13.5 (13.6)	452.1 (211.5‐811.2)	413.7 (199.1‐786.4)	−38.4 (8.5)	92.1 (75.4‐110.6)	79.1 (64.1‐96.9)	−13.0 (14.1)
5‐24	10.2 (7.7‐13.4)	8.7 (6.3‐11.5)	−1.5 (14.8)	67.7 (44.6‐101.8)	61.2 (38.0‐91.9)	−6.5 (9.6)	6.0 (4.1‐8.6)	4.9 (3.0‐7.2)	−1.1 (19.1)
25‐44	14.6 (11.1‐18.7)	13.1 (9.9‐17.1)	−1.5 (10.3)	44.3 (33.3‐57.2)	40.1 (29.9‐52.8)	−4.2 (9.3)	1.7 (0.6‐4.0)	1.3 (0.4‐3.5)	−0.4 (21.5)
45‐64	17.5 (12.2‐24.4)	15.4 (10.5‐22.0)	−2.1 (12.0)	54.6 (33.8‐83.4)	49.0 (29.7‐77.1)	−5.6 (10.1)	8.7 (4.7‐14.6)	7.4 (3.8‐12.9)	−1.3 (14.8)
≥65	40.8 (25.2‐62.3)	37.3 (22.2‐57.8)	−3.5 (8.7)	265.9 (67.9‐638.1)	248.6 (61.3‐603.2)	−17.3 (6.5)	33.6 (19.8‐54.5)	30.5 (16.8‐49.5)	−3.1 (9.2)
<5	153.3 (134.4‐174.3)	136.9 (119.0‐156.9)	−16.4 (10.7)	468.0 (245.5‐829.8)	429.4 (217.4‐779.2)	−38.6 (8.3)	148.0 (129.1‐168.8)	132.0 (114.2‐151.7)	−16.0 (10.8)
≥5	14.2 (12.2‐16.6)	12.6 (10.6‐14.7)	−1.6 (11.9)	53.1 (43.5‐64.2)	48.1 (38.9‐58.7)	−5.0 (9.4)	6.5 (5.0‐8.3)	5.5 (4.1‐7.1)	−1.0 (15.8)
All	29.9 (27.0‐32.9)	26.5 (23.9‐29.4)	−3.4 (11.2)	57.2 (47.4‐68.6)	51.9 (42.6‐62.8)	−5.3 (9.3)	25.0 (22.2‐28.1)	22.0 (19.3‐24.9)	−3.0 (12.0)
Severe Chronic respiratory illness (SCRI‐7)									
<1	99.0 (66.4‐140.5)	97.0 (61.3‐136.7)	−2.0 (2.0)	124.8 (4.1‐289.6)	123.5 (3.5‐281.1)	−1.3 (1.1)	98.7 (66.9‐141.7)	96.8 (64.3‐137.8)	−1.9 (2.0)
1‐4	20.3 (13.2‐30.2)	18.2 (11.3‐27.3)	−2.1 (10.4)	98.1 (103.2‐356.0)	88.8 (10.7‐318.7)	−9.3 (9.5)	18.8 (12.2‐28.8)	16.8 (10.2‐25.8)	−2.0 (10.5)
5‐24	10.2 (7.6‐13.2)	8.8 (6.5‐11.7)	−1.4 (13.3)	40.5 (23.2‐68.2)	37.0 (19.1‐61.3)	−3.5 (8.6)	8.0 (5.7‐10.9)	6.8 4.7‐9.5)	−1.2 (15.1)
25‐44	65.9 (58.4‐74.2)	57.7 (50.8‐65.6)	−8.2 (12.3)	191.1 (168.0‐217.0)	172.4 (150.3‐196.8)	−18.7 (9.8)	11.3 (7.8‐15.9)	7.8 (5.0‐11.8)	−3.5 (30.9)
45‐64	92.7 (79.7‐106.9)	82.7 (70.5‐96.2)	−10.0 (10.8)	363.0 (305.9‐429.2)	329.2 (275.0‐392.5)	−33.8 (9.3)	28.2 (20.3‐37.3)	23.8 (16.7‐32.3)	−4.4 (15.4)
≥65	153.1 (121.4‐191.1)	136.9 (107.6‐173.8)	−16.2 (10.5)	867.6 (476.9‐1463.5)	790.4 (431.3‐1385.1)	−77.2 (8.9)	130.1 (100.5‐165.9)	115.9 (88.2‐150.2)	−14.2 (10.9)
<5	36.0 (27.2‐46.9)	33.9 (25.5‐44.7)	−2.1 (5.8)	101.1 (24.5‐347.1)	92.6 (9.5‐286.1)	−8.5 (8.4)	34.9 (25.9‐45.5)	32.9 (24.2‐43.2)	−2.0 (5.7)
≥5	49.3 (45.4‐53.5)	43.6 (39.9‐47.5)	−5.7 (11.7)	205.8 (186.4‐226.7)	186.1 (167.8‐206.2)	−19.7 (9.6)	18.2 (15.7‐21.1)	15.2 (12.9‐17.8)	−3.0 (16.5)
All	47.8 (44.2‐51.6)	42.5 (39.9‐47.5)	−5.3 (11.2)	200.9 (182.2‐221.4)	181.7 (163.7‐201.0)	−19.2 (9.5)	20.5 (17.9‐23.2)	17.6 (15.2‐20.1)	−2.9 (14.1)

CI, confidence intervals; HIV, human immunodeficiency virus.

a Rates expressed per 100,000 population.

b Estimated rates without adjustment for the attributable fraction.

c Estimated rates adjusted by the attributable fraction.

d Unadjusted minus adjusted rates.

#### 3.2.2 Effects of the INF‐AF on the estimated relative risk associated with HIV infection

Overall the unadjusted RR of influenza‐associated illness due to HIV infection was 2.3 (95% CI: 2.2‐2.4) among ILI cases, 9.7 (95% CI: 8.0‐11.8) among SARI‐10 cases, and 10.0 (95% CI: 8.1‐13.7) among SCRI‐10 cases (Table 5). Across syndromes, this RR was lowest in the <1 year age group (ILI: 1.1; 95% CI: 0.4‐3.1; SARI‐10: 1.5; 95% CI: 0.3‐6.7; SCRI‐10: 2.5; 95% CI: 0.1‐142.3) and highest in the 25‐44 year age group (ILI: 3.4; 95% CI: 3.2‐3.6; SARI‐10: 14.5; 95% CI: 9.5‐21.9; SCRI‐10: 26.6; 95% CI: 14.6‐48.5). After adjusting for the INF‐AF the percent increase (PI) from the overall unadjusted RR was 6.0% (AF‐adjusted RR: 2.4), 15.4% (AF‐adjusted RR: 10.4) and 16.4% (AF‐adjusted RR: 10.5) among ILI, SARI‐10, and SCRI‐10 cases, respectively. There were minimal differences between the unadjusted and AF‐adjusted RR across syndromes and age groups. The effects of the INF‐AF on the RR were highest in the 25‐44 year age group across syndromes (Table 5).

**Table 5:** Estimated relative risk (RR) associated with HIV infection for influenza‐associated influenza‐like illness, severe acute respiratory illness (symptom duration ≤10 or ≤7 days), and severe chronic respiratory illness (symptom duration >10 or >7 days), Klerksdorp and Pietermaritzburg, South Africa, 2013‐2015

Influenza‐associated respiratory illness RR^a^ (95% CI)						
Age group (in years)	Symptoms duration cutoff: 10 days			Symptoms duration cutoff: 7 days		
	Unadjusted^b^	AF‐adjusted^c^	RR % increase^d^	Unadjusted^b^	AF‐adjusted^c^	RR % increase^d^
Influenza‐like illness						
<1	1.1 (0.4‐3.1)	1.2 (0.4‐3.2)	4.6	N/A	N/A	N/A
1‐4	1.1 (0.8‐1.5)	1.2 (0.8‐1.6)	4.8	N/A	N/A	N/A
5‐24	1.4 (1.3‐1.5)	1.5 (1.4‐1.6)	4.1	N/A	N/A	N/A
25‐44	3.4 (3.2‐3.6)	3.7 (3.5‐3.9)	8.9	N/A	N/A	N/A
45‐64	2.6 (2.3‐2.9)	2.7 (2.4‐3.0)	4.2	N/A	N/A	N/A
≥65	2.2 (0.9‐4.8)	2.3 (1.1‐5.1)	4.9	N/A	N/A	N/A
<5^e^	1.1 (0.8‐1.5)	1.1 (0.8‐1.6)	4.4	N/A	N/A	N/A
≥5^f^	2.4 (2.3‐2.5)	2.5 (2.4‐2.6)	6.2	N/A	N/A	N/A
All^g^	2.3 (2.2‐2.4)	2.4 (2.3‐2.6)	6.0	N/A	N/A	N/A
Severe acute respiratory illness						
<1	1.5 (0.3‐6.7)	1.6 (0.3‐7.9)	1.2	1.6 (0.3‐7.7)	1.6 (0.3‐8.3)	0.9
1‐4	4.7 (2.5‐8.7)	4.9 (2.5‐9.4)	4.3	4.9 (2.6‐9.3)	5.2 (2.7‐10.2)	6.5
5‐24	10.3 (6.1‐17.4)	11.8 (6.7‐20.9)	15.0	11.3 (6.6‐19.2)	12.5 (7.0‐22.2)	10.7
25‐44	14.5 (9.5‐21.9)	17.1 (10.7‐27.3)	18.4	26.1 (10.4‐65.3)	30.8 (10.9‐87.6)	18.4
45‐64	14.1 (9.3‐21.6)	15.7 (9.9‐24.8)	6.5	6.3 (3.2‐12.3)	6.6 (3.2‐13.7)	5.5
≥65	7.8 (3.8‐15.8)	7.8 (3.7‐16.5)	0.6	7.9 (2.7‐22.9)	8.2 (2.7‐24.5)	3.0
<5^e^	3.6 (2.0‐6.3)	3.6 (2.0‐6.6)	4.1	3.8 (2.1‐6.8)	3.9 (2.1‐7.3)	6.1
≥5^f^	12.5 (9.9‐15.8)	13.8 (10.8‐17.7)	15.5	11.1 (7.9‐15.6)	12.1 (8.4‐17.4)	15.2
All^g^	9.7 (8.0‐11.8)	10.4 (8.5‐12.7)	15.4	7.9 (6.1‐10.3)	8.4 (6.4‐11.1)	14.9
Severe chronic respiratory illness						
<1	2.5 (0.1‐142.3)	2.5 (0.1‐183.0)	0.9	1.3 (0.1‐39.1)	1.3 9 (0.1‐40.2)	0.9
1‐4	8.1 (1.6‐40.6)	8.6 (1.5‐47.7)	5.4	5.2 (1.3‐20.7)	5.3 (1.2‐22.5)	1.3
5‐24	5.4 (2.9‐9.9)	5.9 (3.0‐11.5)	8.9	5.1 (2.8‐9.2)	5.4 (2.9‐10.2)	7.5
25‐44	26.6 (14.6‐48.5)	32.2 (15.8‐65.5)	20.8	16.9 (11.8‐24.3)	22.1 (14.3‐34.0)	30.7
45‐64	8.4 (5.5‐12.9)	8.5 (5.3‐13.6)	1.5	12.9 (9.2‐18.0)	13.8 (9.6‐19.8)	7.5
≥65	6.2 (3.0‐12.8)	6.3 (2.9‐13.6)	1.9	6.7 (3.7‐11.9)	6.8 (3.7‐12.5)	2.3
<5^e^	6.3 (1.4‐27.3)	6.5 (1.4‐31.1)	5.1	3.6 (1.1‐13.0)	3.6 (1.1‐13.5)	1.3
≥5^f^	10.2 (7.9‐13.0)	10.7 (8.2‐14.0)	17.1	11.4 (9.4‐13.8)	12.6 (10.3‐15.6)	20.9
All^g^	10.0 (7.9‐12.7)	10.5 (8.1‐13.7)	16.4	10.9 (9.1‐13.2)	12.0 (9.8‐14.7)	20.1

RR, relative risk; CI, confidence intervals; AF, attributable fraction.

a HIV‐infected vs HIV‐uninfected

b Estimated relative risk without adjustment for the attributable fraction.

c Estimated relative risk adjusted by the attributable fraction.

d Attributable fraction‐adjusted vs unadjusted relative risk.

e RR and RR percent increase adjusted by age with the following categories: <1 and 1‐4 years.

f RR and RR percent increase adjusted by age with the following categories: 5‐24, 25‐44, 45‐64, and ≥65 years.

g RR and RR percent increase adjusted by age with the following categories: <1, 1‐4, 5‐24, 25‐44, 45‐64, and ≥65 years.

The effects of the AF of influenza virus infection to illness on the estimated RR among SARI‐7 and SCRI‐7 cases were similar to those observed among SARI‐10 and SCRI‐10 cases (Table 5). The unadjusted RR and AF‐adjusted RR of influenza‐associated illness due to HIV infection among any hospitalized patient are provided in Table S1.

### 3.3 Effects of the duration of symptoms on burden estimates of influenza‐associated severe respiratory illness

**Figure 3:**
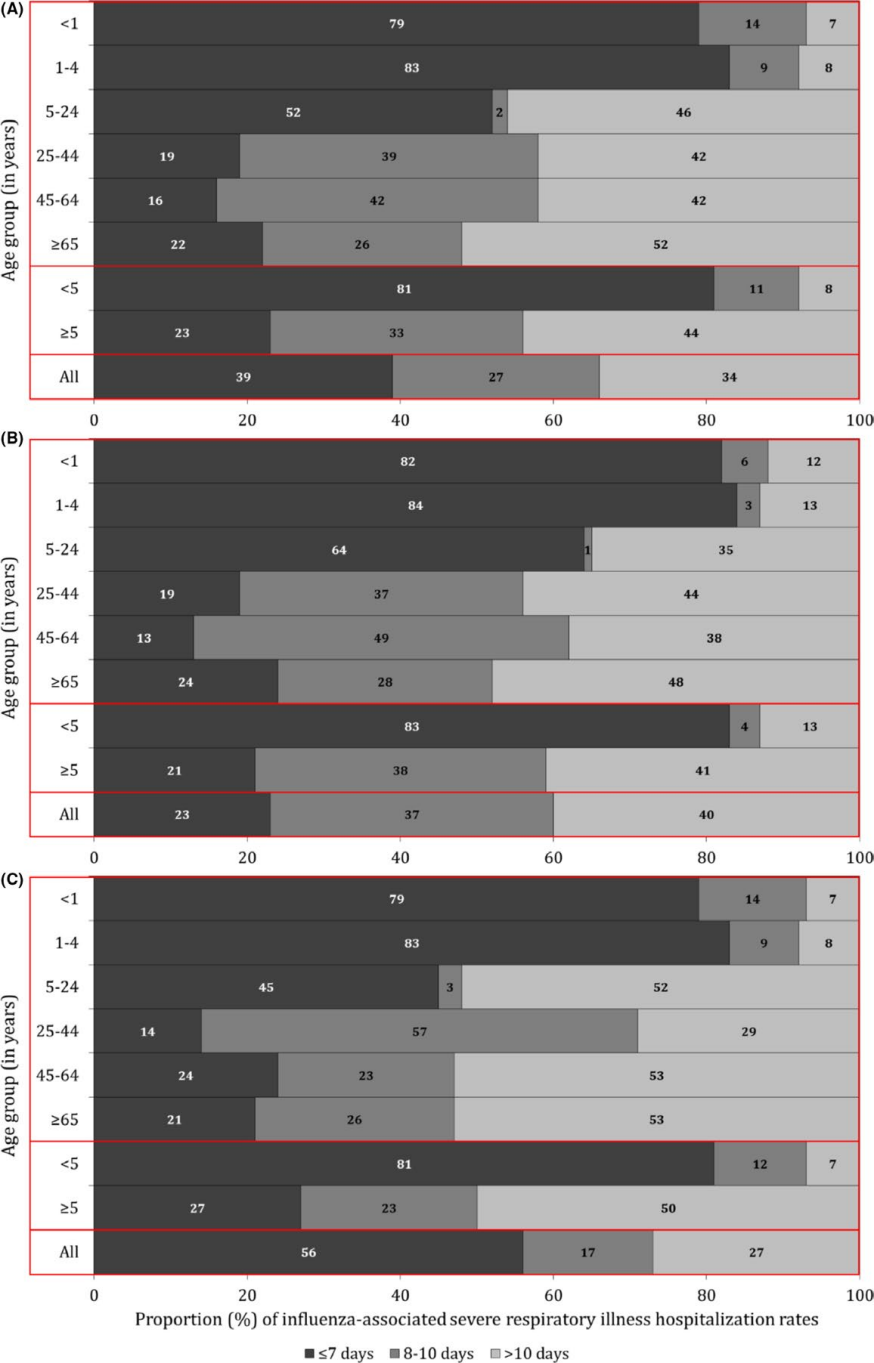
Proportion of attributable fraction‐adjusted influenza‐associated severe respiratory illness hospitalization rates by duration of symptoms, Klerksdorp and Pietermaritzburg, South Africa, 2013‐2015. (A) All; (B) HIV‐infected; (C) HIV‐uninfected

Overall 39% of the AF‐adjusted influenza‐associated severe respiratory illness hospitalizations had symptom duration of ≤7 days prior to admission (Figure 3 panel A). An additional 27% had symptom duration of 8‐10 days, and the remaining 34% had symptom duration of >10 days. Among children aged <5 years, 81% had symptom duration of ≤7 days and only 8% presented at the hospital >10 days after symptom onset. On the contrary, only 23% of individuals aged ≥5 years presented to the hospital with symptom onset ≤7 days, whereas 44% had symptoms duration of >10 days. A similar pattern was observed among HIV‐infected and HIV‐uninfected individuals (Figure 3 panels B and C).

## 4 DISCUSSION

We described the effects of the INF‐AF and duration of symptoms on influenza disease burden estimates among HIV‐infected and HIV‐uninfected patients of different age groups with mild or severe respiratory illness. A minimal difference was observed between unadjusted and AF‐adjusted rates or RR associated with HIV infection across syndromes, age groups, and HIV serostatus. Whereas shifting the symptom duration cutoff for inclusion in SARI surveillance from ≤7 to ≤10 days resulted in an overall 27% increase in estimated influenza‐associated hospitalization rates, 34% of patients with influenza‐associated severe respiratory illness of any duration had symptom duration >10 days. The latter would have been missed by applying the symptom duration cutoff of ≤10 days suggested by WHO. The proportion of cases that could have been missed by applying the ≤10 symptom duration cutoff was higher (44%) among individuals aged ≥5 years and lower among children aged <5 years (8%). A similar pattern was observed among HIV‐infected and HIV‐uninfected individuals. Persons aged <5 years and ≥65 years and HIV‐infected individuals experienced elevated rates of influenza‐associated respiratory illness hospitalization.

The minimal difference between unadjusted and AF‐adjusted rates observed in this study is likely a reflection of the high INF‐AF used for adjustment in this study (ILI: 92.6%; SARI‐10: 87.4%; SCRI‐10: 86.2%).^12^

HIV‐infected patients were at increased risk of influenza‐associated mild or severe illness compared to HIV‐uninfected patients. HIV infection has been reported to be associated with increased risk of influenza‐associated SARI hospitalization in other studies.^4^, ^23^, ^24^ The RR of HIV infection to illness among influenza‐positive patients with ILI or SCRI has not been described to our knowledge.

The magnitude of the INF‐AF used for adjustment in this study was highest among HIV‐uninfected persons aged <1 and ≥65 years and lowest among persons aged 25‐44 years for all syndromes, but not among HIV‐infected patients whereby the magnitude of the INF‐AF was similar across age groups for all syndromes.^12^ We observed the highest percent increase in the AF‐adjusted compared to unadjusted RR due to HIV infection among individuals aged 25‐44 years. This is due to the highest difference of the magnitude of the INF‐AF between HIV‐infected and HIV‐uninfected individuals in this age group.^12^ However, the percent increase in the AF‐adjusted compared to unadjusted RR due to HIV infection was minimal across age groups and syndromes.

In our study, only 8% of children aged <5 years presented with symptom duration >10 days compared to 44% among individuals aged ≥5 years. This may be due to differential healthcare‐seeking behavior across different age groups whereby young children are brought for medical attention at the earlier stages of illness compared to older individuals. Nonetheless, it should be noted that the SARI case definition used in this study among children aged <5 years is broader than those recommended by WHO. Among influenza‐positive children aged <5 years hospitalized with any severe respiratory illness, 68.1% met the WHO SARI case definition; 57.5% among infants aged <1 year and 79.6% among children aged 1‐4 years, respectively. This suggests that by applying the current WHO SARI case definition a high percentage of influenza‐associated severe respiratory illness would have been missed also among young children. In young children, the use of fever and cough as inclusion criteria for SARI surveillance as recommended by WHO may play more of an important role in the sensitivity of the case definition compared to symptom duration; however, this was not explicitly explored in this analysis.

In our study, individuals aged 5‐24 years experienced the highest rates of influenza‐associated ILI. This was similar to a study conducted in the United States whereby individuals aged 2‐17 years experienced the highest rates of influenza‐associated medically attended ILI,^25^ suggesting the importance of this age group in the transmission of influenza viruses.^26^ We observed a U‐shaped trend of the magnitude of the influenza‐associated SARI and SCRI hospitalization rates across age groups. Young children and older individuals are known to be at increased risk of influenza‐associated severe illness.^4^, ^23^ In our study, the highest rates of influenza‐associated SARI hospitalization were among children aged <1 year, whereas the highest rates of influenza‐associated SCRI hospitalization were among individuals aged ≥65 years. This suggests that using the recommended WHO SARI case definition may underestimate the burden of influenza‐associated severe illness particularly in older individuals in our setting.

Our study has limitations that warrant discussion. First, we did not enroll ILI cases with symptoms duration >10 days hindering our ability to estimate the burden of influenza‐associated medically attended ILI in this group of patients. Second, whereas we enrolled patients with respiratory illness of any duration, patients not meeting the clinical inclusion criteria may have been missed, especially in adults. In addition, we evaluated only the effects of duration of symptoms on disease burden estimates, whereas effects based on other clinical inclusion criteria (such as the presence of fever or cough) were not investigated. Third, whereas PRC is considered a sensitive diagnostic test, the sensitivity of the assay may be lower in individuals presenting at healthcare facilities several days post‐infection. This may underestimate the burden of influenza‐associated illness in this group of patients. Last, ecological studies have suggested that influenza viruses are also responsible for hospitalizations and deaths among patients presenting with circulatory illnesses or even syndromes other than respiratory and circulatory.^5^, ^6^, ^8^, ^9^, ^27^ In addition, individuals that may have developed influenza‐associated severe illness, but did not seek care would have been missed in our study. Hence, our estimates should be considered minimum estimates focusing only on influenza‐associated medically attended respiratory illness.

In conclusion, the AF‐adjusted rates and RR due to HIV infection reported in this study reflect a more accurate description of the burden of influenza‐associated mild and severe respiratory illness among HIV‐infected and HIV‐uninfected South African patients. Nonetheless, the marginal differences between unadjusted and AF‐adjusted rates and RR due to HIV infection are unlikely to affect policies or prioritization of interventions. On the contrary, the use of the duration of symptoms included in the WHO SARI case definition may importantly underestimate influenza‐associated severe disease burden in both children and adults in our settings. The use of such surveillance case definition for disease burden estimation should be evaluated in different settings, especially in low‐ and middle‐income countries where cultural differences and limited access to care may affect the time of presentation at healthcare facilities among patients seeking medical attention.

## DISCLAIMER

The findings and conclusions in this report are those of the authors and do not necessarily represent the official position of the US Centers for Disease Control and Prevention, USA, or the National Institute for Communicable Diseases, South Africa.

## COMPETING INTERESTS

All authors declare that they have no commercial or other associations that may pose a conflict of interest.

## FINANCIAL DISCLOSURE

This work was supported by the National Institute for Communicable Diseases, of the National Health Laboratory Service and the US Centers for Disease Control and Prevention (co‐operative agreement number: 5U51IP000155).

## ETHICS

The SCRI and SARI protocols were approved by the University of the Witwatersrand Human Research Ethics Committee (HREC) and the University of KwaZulu‐Natal Human Biomedical Research Ethics Committee (BREC) protocol numbers M081042 and BF157/08, respectively. The ILI and controls protocol were approved by BREC protocol number (BREC BF 080/12). This surveillance was deemed non‐research by the US Centers for Disease Control and Prevention (non‐research determination number: 2012‐6197).

## AUTHOR CONTRIBUTIONS

All authors had full access to the data in the study and take responsibility for the integrity of the data and the accuracy of the data analysis. Tempia S and Cohen C. involved in study concept and design. Tempia S, Walaza S, Moyes J, Cohen AL, von Mollendorf C, Treurnicht F, Venter M, Pretorius M, Hellferscee O, Nguweneza A, Wolter N, von Gottberg A, Dawood H, Variava E, and Cohen C. involved in acquisition, analysis or interpretation of data. Tempia S. involved in drafting of the manuscript. Tempia S, Walaza S, Moyes J, Cohen AL, von Mollendorf C, McMorrow ML, Mhlanga S, Teurnicht FK, Venter M, Pretorius M, Hellferscee O, Nguweneza A, McAnerney JM, Wolter N, von Gottberg A, Dawood H, Variava E, Madhi SA and Cohen C involved in critical revision of the manuscript for important intellectual content.
