# Supplementary material for: The effects of the attributable fraction and the duration of symptoms on burden estimates of influenza‐associated respiratory illnesses in a high HIV prevalence setting, South Africa, 2013‐2015
Source: Influenza Other Respir Viruses. 2018 Feb 1;12(3):360–73. doi: 10.1111/irv.12529 (PMC5907815; doi:10.1111/irv.12529)
Supplement: Supplementary file 1 [file IRV-12-360-s001.docx]

**The Effects of the Attributable Fraction and the Duration of Symptoms on Burden Estimates of Influenza-Associated Respiratory Illnesses in a High HIV-Prevalence Setting, South Africa, 2013-2015 (Supplementary Material)**

**Authors**

Stefano Tempia^1,2,3*^, Sibongile Walaza^3,4^, Jocelyn Moyes^3,4^, Adam L. Cohen^1,5^, Claire von Mollendorf^3,4^, Meredith L. McMorrow^1,2^, Sarona Mhlanga^3^, Florette K. Treurnicht^3^, Marietjie Venter^6^, Marthi Pretorius^3,6,7^, Orienka Hellferscee^3,8^, Nicole Wolter^3,8^, Anne von Gottberg^3,8^, Arthemon Nguweneza^3^, Johanna M. McAnerney^3^, Halima Dawood^9,10^, Ebrahim Variava^11,12,13^, Shabir A. Madhi^3,14,15^, Cheryl Cohen^3,4^

**Affiliations**

^1^ Influenza Division, Centers for Disease Control and Prevention, Atlanta, Georgia, United States of America.

^2^ Influenza Program, Centers for Disease Control and Prevention, Pretoria, South Africa.

^3^ Centre for Respiratory Diseases and Meningitis, National Institute for Communicable Diseases of the National Health Laboratory Service, Johannesburg, South Africa.

^4^ School of Public Health, Faculty of Health Sciences, University of the Witwatersrand, Johannesburg, South Africa.

^5^ Global Immunization Monitoring and Surveillance Team, Expanded Programme on Immunization, Department of Immunization, Vaccines and Biological, World Health Organization, Geneva, Switzerland.

^6^ Centre for Viral Zoonoses, Department of Medical Virology, University of Pretoria, Pretoria, South Africa.

^7^ Tshwane Academic Division, National Health Laboratory Service, Pretoria, South Africa.

^8^ School of Pathology, Faculty of Health Sciences, University of the Witwatersrand, Johannesburg, South Africa.

^9^ Department of Medicine, Pietermaritzburg Metropolitan Hospital, Pietermaritzburg, South Africa.

^10^ Department of Medicine, University of KwaZulu-Natal, Pietermaritzburg, South Africa.

^11^ Department of Medicine, Klerksdorp-Tshepong Hospital Complex, Klerksdorp, South Africa.

^12^ Department of Medicine, Faculty of Health Sciences, University of the Witwatersrand, Johannesburg, South Africa.

^13^ Perinatal HIV Research Unit, University of the Witwatersrand, Johannesburg, South Africa.

^14^ Medical Research Council, Respiratory and Meningeal Pathogens Research Unit, University of the Witwatersrand, Johannesburg, South Africa.

^15^ Department of Science and Technology/National Research Foundation: Vaccine Preventable Diseases, University of the Witwatersrand, Johannesburg, South Africa.

**METHODS**

**Statistical analysis**

*Rates of influenza-associated respiratory hospitalizations and outpatient consultations*

The equation used to estimate the rates of influenza-associated SARI (used for either SARI-10 or SARI-7 cases) hospitalization is provided below:

$Infl_{SARIRate_{i}}=\frac{SARI_{Enrolled_{i}}*\frac{7}{5}*\frac{1}{X_{SARI_{i}}}*\frac{1}{HUS_{SARI}}*Infl_{SARIDetectRateObs_{i}}*Infl_{SARIAF_{i}}}{Pop_{i}}*100,000$ (1)

Where $Infl_{SARIRate_{i}}$ is the estimated age-specific rate of influenza-associated SARI hospitalization in age group *i*; $SARI_{Enrolled_{i}}$is the age-specific number of SARI cases enrolled; 7/5 is the coefficient used to adjust for non-enrolment over weekends; $X_{SARI_{i}}$ is the age-specific proportion of all eligible SARI cases that were enrolled (obtained from study logs); *HUS_SARI_* is the proportion of SARI cases that sought care at the surveillance site over the total number of SARI cases that sought care to any hospital obtained from healthcare utilization surveys (HUS) conducted in the catchment areas of the surveillance sites [[[1]](#endnote-1),[[2]](#endnote-2)]; $Infl_{SARIDetectRateObs_{i}}$is the observed age-specific influenza detection rate among SARI cases tested; $Infl_{SARIAF_{i}}$is the age-specific influenza-virus AF among SARI cases estimated at the same sentinel sites during the same study period [[[3]](#endnote-3)]; and *Pop_i_* is the age-specific mid-year population at risk [[[4]](#endnote-4)]. For this analysis we assumed that the number of admission during weekends was the same as during working days as reported in previous studies conducted in South Africa [[[5]](#endnote-5)].

The same approach was used to estimate the rates of influenza-associated SCRI (used for either SCRI-10 or SCRI-7 cases) hospitalizations.

The equation used to estimate the rates of influenza-associated ILI consultations is provided below:

$Infl_{ILIRate_{i}}=SARI_{Rate_{i}}*Z*X*\frac{Y_{ILI_{i}}}{Y_{SARI_{i}}}*Infl_{ILIDetectRateObs_{i}}*Infl_{ILIAF_{i}}*100,000$ (2)

Where $Infl_{ILIRate_{i}}$ is the estimated age-specific rate of influenza-associated ILI outpatient consultations in age group *i*; $SARI_{Rate_{i}}$is the age specific rate of SARI-10 hospitalization (adjusted for non-enrollment and healthcare seeking behavior as obtained in equation 1); *Z* is the proportion of SARI-10 cases that sought outpatient care before hospitalization; *X* is ratio of ILI consultation referred to hospital to the total number of ILI consultations obtained from ILI surveillance over the study period; $Y_{ILI_{i}}$ is the proportion of ILI cases in age group *i* over the total number of ILI cases after adjusting for non-enrolment; $Y_{SARI_{i}}$ is the proportion of SARI cases in age group *i* over the total number of SARI cases after adjusting for non-enrolment; $Infl_{ILIDetectRateObs_{i}}$is the observed age-specific influenza detection rate among ILI cases tested; and $Infl_{ILIAF_{i}}$ is the age-specific influenza virus AF among ILI cases estimated at the same sentinel sites within the study period [3].

**RESULTS**

**Table S1: Estimated mean annual rates of and relative risk associated with HIV-infection for influenza-associated severe respiratory illness hospitalization (any duration of symptoms), Klerksdorp and Pietermaritzburg, South Africa, 2013-2015.**

| **Age group**  **(in years)** | **Influenza-associated severe respiratory illness hospitalization rates^a^**  **(95% CI)** | | | **RR HIV-infected vs. HIV-uninfected**  **(95% CI)** |
| --- | --- | --- | --- | --- |
|  | **All** | **HIV-infected** | **HIV-uninfected** |  |
| **Unadjusted^b^** | | | | |
| <1 | 470.6 (395.4-552.7) | 720.4 (91.7-2736.6) | 468.4 (395.8-553.9) | 1.6 (0.4-6.5) |
| 1-4 | 119.0 (101.0-140.6) | 550.2 (305.3-980.6) | 110.9 (92.9-131.6) | 4.7 (2.6-8.6) |
| 5-24 | 20.4 (15.7-24.6) | 108.2 (78.8-150.3) | 14.0 (10.9-17.7) | 7.7 (5.2-11.3) |
| 25-44 | 80.5 (72.2-89.6) | 235.3 (208.7-262.8) | 12.9 (9.2-17.9) | 18.1 (12.9-25.3) |
| 45-64 | 110.2 (96.1-125.7) | 417.6 (356.1-488.1) | 36.8 (27.8-47.2) | 11.4 (8.5-15.4) |
| ≥65 | 193.9 (157.9-236.0) | 1133.6 (664.7-1772.4) | 163.7 (130.6-203.9) | 6.8 (4.1-11.3) |
| <5^d^ | 189.3 (168.2-212.6) | 569.1 (303.0-929.9) | 182.9 (161.6-205.6) | 3.7 (2.2-6.3) |
| ≥5^e^ | 63.6 (59.2-68.3) | 258.9 (237.0-282.2) | 24.7 (21.8-28.1) | 11.3 (9.6-13.4) |
| All^f^ | 77.7 (73.1-82.6) | 258.2 (236.0-280.8) | 45.5 (41.6-49.5) | 9.8 (8.4-11.4) |
| **AF-adjusted^c^** | | | | |
| <1 | 436.8 (365.3-517.1) | 674.6 (11.6-2136.6) | 434.7 (361.6-501.2) | 1.7 (0.4-7.0) |
| 1-4 | 103.1 (85.9-122.9) | 494.9 (273.5-924.6) | 95.7 (78.5-114.3) | 5.1 (2.7-9.4) |
| 5-24 | 16.8 (13.5-20.7) | 95.9 (64.9-131.1) | 11.0 (8.3-14.4) | 8.7 (5.7-13.4) |
| 25-44 | 69.3 (61.5-77.7) | 206.6 (182.8-233.7) | 9.6 (6.4-13.9) | 21.5 (14.5-31.7) |
| 45-64 | 95.5 (82.1-109.6) | 366.5 (308.3-431.9) | 30.7 (22.5-40.2) | 11.8 (8.6-16.3) |
| ≥65 | 173.7 (140.4-214.7) | 1027.3 (617.1-1695.8) | 146.3 (114.6-183.8) | 6.8 (4.0-11.7) |
| <5^d^ | 169.7 (149.7-191.7) | 514.7 (274.2-880.1) | 164.0 (144.1-185.8) | 3.9 (2.2-6.8) |
| ≥5^e^ | 54.8 (50.7-59.1) | 227.7 (206.8-249.2) | 20.5 (17.8-23.4) | 12.2 (10.2-14.7) |
| All^f^ | 67.7 (63.4-72.2) | 227.2 (207.2-248.9) | 39.2 (35.6-42.9) | 10.4 (8.8-12.2) |

Abbreviations: CI: confidence intervals; RR: relative risk; HIV: human immunodeficiency virus.

^a^ Rates expressed per 100,000 population.

^b^ Estimated rates without adjustment for the attributable fraction.

^c^ Estimated rates adjusted by the attributable fraction.

^d^ Relative risk adjusted by age within the following categories: <1 and 1-4 years.

^e^ Relative risk adjusted by age within the following categories: 5-24, 25-44, 45-64 and ≥65 years.

^f^ Relative risk adjusted by age within the following categories: <1, 1-4, 5-24, 25-44, 45-64 and ≥65 years.

**Figure S1: HIV prevalence among patients with mild or severe respiratory illness, Klerksdorp and Pietermaritzburg, South Africa, 2013-2015. A: Influenza-like illness; B: Severe acute respiratory illness (symptom duration ≤10 days); C: Severe chronic respiratory illness (symptom duration >10 days).**

**Figure S2: Overall unadjusted and AF-adjusted mean annual influenza-associated severe respiratory illness hospitalization rates (any duration of symptoms) by age group, Klerksdorp and Pietermaritzburg, South Africa, 2013-2015.**

**REFERENCES**

1. Wong KK, von Mollendorf C, Martinson NA, et al. Healthcare utilization for common infectious disease syndromes in Soweto and Klerksdorp, South Africa. [↑](#endnote-ref-1)
2. McAnerney JM, Cohen C, Cohen AL, et al. Healthcare utilization patterns for common syndromes in Pietermaritzburg, KwaZulu-Natal Province, South Africa, 2013. [↑](#endnote-ref-2)
3. Tempia S, Walaza S, Moyes J, et al. Attributable fraction of influenza virus detection to mild and severe respiratory illness in HIV-infected and HIV-uninfected patients, South Africa, 2012-2016. Emerg Infect Dis. **2017**; 23(7):1124-1132. [↑](#endnote-ref-3)
4. Statistics South Africa – 2011 Census. Available at: <http://www.statssa.gov.za/?page_id=3839>. Accessed on 25 July 2016. [↑](#endnote-ref-4)
5. Cohen C, Moyes J, Tempia S, et al. Severe influenza-associated lower respiratory tract infection in a high HIV-Prevalence setting – South Africa, 2009-2011. Emerg Infec Dis. **2013**; 19(11):1766-74. [↑](#endnote-ref-5)
